# Effect of Ferrule Height on the Fracture Resistance of Endodontically Treated Teeth Restored With Glass Fiber Posts: An In Vitro Study

**DOI:** 10.7759/cureus.79583

**Published:** 2025-02-24

**Authors:** Sneha Rathaur, Pankaj K Gupta, Sonal Dhote, Kumari S Pravin, Komal Kishlay, Seema Gupta

**Affiliations:** 1 Department of Conservative Dentistry and Endodontics, Rungta College of Dental Sciences and Research, Bhilai, IND; 2 Department of Prosthodontics, Aditi Dental Clinic and Implant Centre, Patna, IND; 3 Department of Prosthodontics, Sarjug Dental College and Hospital, Darbhanga, IND; 4 Department of Orthodontics, Kothiwal Dental College and Research Centre, Moradabad, IND

**Keywords:** endodontically treated teeth, fracture, glass fiber, post, resistance

## Abstract

Introduction: Restoration of endodontically treated teeth (ETT) presents a significant challenge due to compromised structural integrity. The ferrule effect is crucial in reinforcing teeth and improving fracture resistance. This study aimed to compare and evaluate the effect of different crown ferrules on the fracture resistance of ETT restored with a glass fiber post and to evaluate the failure pattern.

Materials and methods: Fifty-six extracted human maxillary central incisors were selected and randomly divided into four groups (n = 14 per group) based on ferrule height: Group 1 (control, no ferrule with a metal post), Group 2 (0 mm ferrule with a glass fiber post), Group 3 (2 mm ferrule with a glass fiber post), and Group 4 (3 mm ferrule with a glass fiber post). After endodontic treatment and post-space preparation, the teeth were restored using composite cores and full metal crowns. A universal testing machine was used to apply a compressive load at a 45° angle until fracture occurred. The fracture resistance was recorded in Newtons (N), and the failure modes were analyzed under a stereomicroscope. Statistical analysis was performed using analysis of variance (ANOVA) and post-hoc Tukey test (p < 0.05).

Results: The mean fracture resistance values were highest in Group 1 (559.68 ± 92.52 N) and lowest in Group 2 (215.60 ± 70.79 N). Group 3 (428.53 ± 154.27 N) and Group 4 (439.06 ± 118.83 N) exhibited significantly higher fracture resistance than Group 2 (p = 0.001) but were not significantly different from each other (p = 0.950). Unfavorable root fractures were predominant in Group 1 and Group 2, whereas Group 3 and Group 4 showed repairable core-debonding failures.

Conclusions: Ferrules play a significant role in the fracture resistance of glass fiber post-restored teeth. Increasing the ferrule length significantly increases fracture resistance. Although metal posts provide higher fracture resistance, they are associated with a greater incidence of non-repairable failures. Glass fiber posts combined with an adequate ferrule are a more favorable option for restoring structurally compromised teeth.

## Introduction

Rehabilitating significantly compromised endodontically treated teeth (ETT) presents a challenge for dental practitioners and necessitates meticulous treatment planning. Different treatment modalities exist, encompassing direct composite restorations, post-and-core restorations, and full-coverage crowns [1]. The post-and-core technique remains the gold standard for reinforcing structurally compromised teeth, particularly when significant coronal dentin is lost [2]. Custom-fabricated metallic posts and cast post-and-core systems have been utilized for an extended period owing to their exceptional compatibility with the prepared post space, substantial retention, and resistance to fracture [3]. Nonetheless, these systems exhibit several drawbacks, such as elevated rigidity, stress concentration at the post-dentin junction, and an increased likelihood of severe root fractures, which frequently require extraction [4].

To overcome these disadvantages, prefabricated glass fiber posts have gained popularity because of their biomechanical compatibility, high flexural strength, and ability to distribute occlusal forces more evenly within the root structure [2]. Unlike metal posts, glass fiber posts have an elastic modulus similar to dentin, reducing stress concentration and the likelihood of vertical root fractures [5]. Additionally, they offer enhanced esthetics, ease of placement, and the ability to bond with adhesive resin cement, thus improving overall retention [6]. However, the long-term success of glass fiber posts is influenced by several factors, including post-design, cementation techniques, bonding effectiveness, core material selection, and, most importantly, the presence of a ferrule [2,7].

The primary concern with ETT restored with post-and-core systems is their increased susceptibility to fractures, primarily due to altered mechanical properties and loss of vital dentin [2]. The lack of a natural dentin structure reduces fracture resistance, making it more prone to failure under occlusal loading [8]. Fracture resistance in ETT is affected by root morphology, post-adaptability, bonding quality, occlusal forces, and restoration design [9]. However, one of the most critical determinants in preventing root fractures in post-restored teeth is the presence of a ferrule [10].

The ferrule effect refers to the circumferential band of the remaining tooth structure that surrounds the post-and-core restoration and extends coronally beyond the margin of the prepared tooth [8]. A properly designed ferrule enhances fracture resistance by providing a bracing or hugging effect, dissipating functional stresses that would otherwise be concentrated at the post-dentin interface. It significantly reduces post-debonding, vertical root fractures, and core failures, thereby improving the long-term prognosis of ETT [11]. Studies have shown that a minimum ferrule height of 1.5-2 mm significantly enhances fracture resistance, with increased resistance observed at greater ferrule heights [12]. The mechanism of action of ferrule lies in its ability to reinforce the remaining coronal dentin, preventing leverage forces that can cause post-dislodgment or root fractures [13,14]. Notwithstanding the acknowledged significance of ferrule, there exists a paucity of scholarly articles investigating the quantitative impact of varying ferrule heights on the fracture resistance of ETT rehabilitated with glass fiber posts. Therefore, this study aimed to compare and evaluate the effect of different crown ferrules on the fracture resistance of ETT restored with a glass fiber post and to evaluate the failure pattern.

## Materials and methods

Study design

The present in vitro study was conducted in the Department of Conservative Dentistry and Endodontics at the Rungta College of Dental Science and Research, Bhilai, from April 2023 to December 2023. Ethical committee approval was waived for this in vitro study, as it utilized extracted teeth collected with the patient's informed consent in accordance with relevant ethical guidelines, and the study followed the principles of the Declaration of Helsinki.

Sample size estimation

Sample size estimation was performed using G*Power software version 3.6.9 (Heinrich-Heine-Universität Düsseldorf, Düsseldorf, Germany) to achieve a statistical power of 80% with an alpha error of 5%. This calculation was based on an effect size of 0.46, as reported in a previous study by Pereira et al. [14], who investigated the fracture resistance of ETT with varying ferrule thickness. These parameters were applied in an analysis of variance (ANOVA) fixed-effect omnibus one-way analysis considering four groups. The a priori computation yielded a total sample size of 56, evenly distributed into three experimental groups and one control group with 14 samples per group, ensuring robust statistical validity.

Methodology

**Figure 1:**
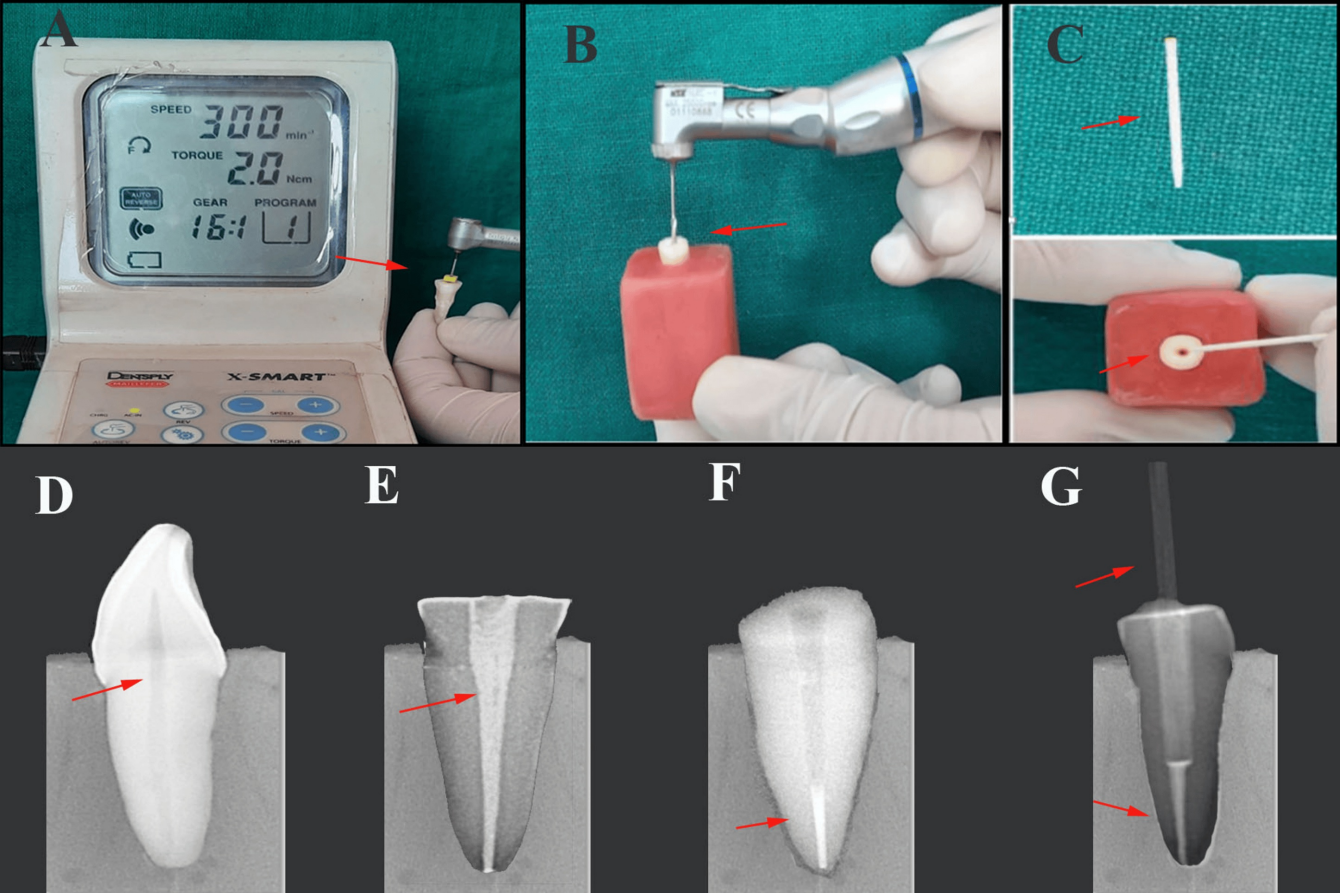
Methodology of post and core: (A) root canal preparation, (B) post-space preparation by micromotor, (C) post space for glass fiber post, (D) selected incisor tooth for post and core, (E) obturated canal with sealer, (F) post space visible on radiograph, and (G) fiber post cemented in canal Figure is derived from the study data.

Fifty-six extracted human permanent maxillary central incisors were included in this study. All teeth were cleaned and stored in 0.1% thymol solution to prevent dehydration. A reference line was marked at the cementoenamel junction (CEJ) on the proximal surface using a graphite pencil based on precise measurements obtained with a digital Vernier caliper. The root canals were accessed, and apical patency was confirmed using the #10 K file (Mani Medical Private Ltd., Delhi, India). A small amount of wax was placed at the root apex to prevent the extrusion of the irrigant solution. Canals were prepared using the crown-down technique with ProTaper rotary nickel-titanium instruments (Mani Medical Private Ltd., Delhi, India). Irrigation and recapitulation were alternated using 2 mL 3% sodium hypochlorite (Ammdent, Punjab, India) and a #10 K-file after each file change. Final irrigation was performed with 5 mL of 17% ethylenediaminetetraacetic acid (Ammdent, Punjab, India) for 1 min, followed by a saline rinse. The canals were dried with absorbent paper points (DiaDent International, Seoul, South Korea) and obturated with F2 gutta-percha (DiaDent International, Seoul, South Korea) using a Dentsply AH Plus Root Canal Sealant (Dentsply Sirona, Munich, Germany). Post-space preparation was performed seven days after obturation. A heated plugger and #3 Peeso reamer (Mani Medical Private Ltd., Delhi, India) removed gutta-percha, leaving a 5 mm apical seal to maintain root integrity (Figure 1).

**Figure 2:**
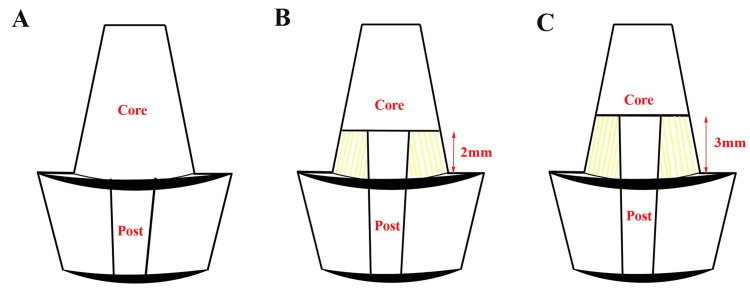
Ferrule preparation in samples: (A) no ferrule at CEJ, (B) 2 mm of ferrule at CEJ, and (C) 3 mm ferrule at CEJ CEJ: cementoenamel junction Figure is the author's original work.

The extracted teeth were randomly divided into three experimental groups (n = 14 each) and one control group (n = 14) based on the ferrule height and post-placement. The control group (Group 1) comprised teeth with no ferrule and were restored with a prefabricated stainless steel metal post (Surtex® Classic Posts, Dentatus, NY, USA). Group 2 included teeth with no ferrule and was restored with a glass fiber post (Angelus, Parana, Brazil). Group 3 had a 2 mm ferrule and was restored with a glass fiber post, while Group 4 featured a 3 mm ferrule with a glass fiber post. The coronal segments of the specified teeth were meticulously sectioned in accordance with the established ferrule height using a diamond disc, ensuring that the incision was perpendicular to the longitudinal axis while employing continuous water irrigation to mitigate the risk of thermal injury. In Group 1 and Group 2, the coronal tooth structure was circumferentially reduced to a flat plane at the CEJ. In Group 3 and Group 4, the coronal crown structures were reduced to a planar configuration at 2 mm and 3 mm incisal elevations to the CEJ, respectively, and restored using the same technique as in the no ferrule cohort (Figure 2).

The core build-up was performed using a Tetric N-Ceram composite resin (Ivoclar, Vivadent, Schaan, Liechtenstein). A preformed polyester matrix was placed on the specimen, and incremental composite layering was performed. All cores were prepared by hand to a 7 mm height. Five increments of the composite resin were applied, each requiring 60 seconds of polymerization to complete the coronal core. A polymerization unit (Ultraled, Dabi Atlante, Ribeirao Preto, São Paulo, Brazil) (110 W) polymerized the composite resin specimens. The tip of the light source was positioned 2 cm from the specimen at the top of the core.

All experimental samples were prepared using diamond rotary cutting instruments under water spray to receive full metal crowns (1.5 mm facial reduction with a chamfer finish line and 0.5 mm chamfered lingual reduction). The finish lines for all the specimens were placed at the CEJ level. Metal copings (Durabond, Sao Paulo, Brazil) of 0.5 mm thickness and 8 mm height were fabricated in the laboratory and used to simulate the final restoration. The crown form was verified using an initial silicone index, and the crowns were luted with type 1 glass ionomer cement (Orikam Healthcare India Pvt. Ltd., Haryana, India).

**Figure 3:**
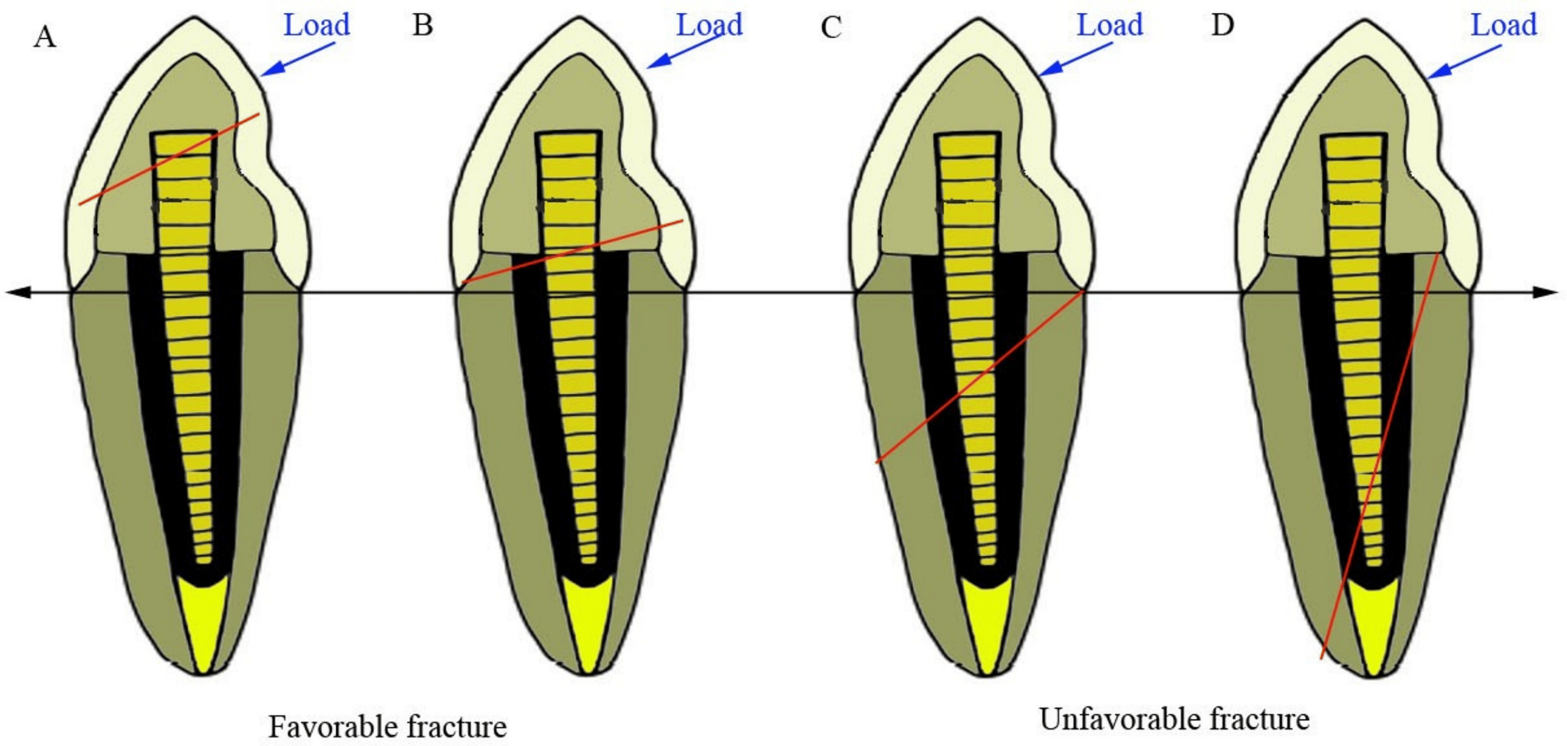
Types of fracture in ETT: (A) core fracture, (B) core and post fracture, (C) coronal third root fracture, and (D) vertical root fracture The fracture line is represented as a red arrow, and the load applied is represented as a blue arrow. ETT: endodontically treated teeth Figure is the author's original work.

Samples were marked 2 mm below the CEJ and covered with 0.6 mm silver foil to simulate the periodontal ligament space. A custom-made rectangular metal mold (2 × 1.5 × 1.5 cm) was used to embed the samples in an autopolymerizing acrylic resin along the long axis. Once set, the silver foil was removed, and a silicone-based impression material was injected into the resin block before reinserting the samples. All specimens were individually mounted on a custom jig positioned at a 45° angle and secured to the lower fixed compartment of a Universal Testing Machine (Metkorp Equipments Pvt. Ltd., Delhi, India). A custom steel rod with a round end (2 mm diameter) was used to apply a compressive load at a crosshead speed of 1 mm/min until fracture occurred. The fracture resistance was recorded in Newtons (N). The type of failure was evaluated using a stereomicroscope at 20x magnification (Biotron Healthcare, India), and fractures were categorized into two types [15]. A favorable (repairable) failure was defined as a fracture occurring above the level of the embedded resin. In contrast, an unfavorable (non-repairable) failure was characterized by a fracture extending below the embedded resin level (Figure 3).

Statistical analysis

Statistical analyses were performed using SPSS Statistics version 23 (IBM Corp. Released 2019. IBM SPSS Statistics for Windows, Version 26.0. Armonk, NY: IBM Corp). All data were tested for normality using the Kolmogorov-Smirnov test, and the data were found to be normally distributed. One-way ANOVA was used to compare fracture resistance among the four groups. Post-hoc analyses were performed using Tukey’s multiple comparison tests to identify significant differences between groups, with a significance threshold set at p < 0.05.

## Results

The distribution of fracture locations after load application among the four groups reveals distinct patterns. Group 1 and Group 2 exhibited a significant proportion of vertical root fractures. In contrast, Group 3 and Group 4 reported predominantly core debonding. Vertical root fractures are severe complications often associated with excessive stress transmission to the root structure. These fractures suggest the poor stress distribution in Group 1 and Group 2. Core debonding indicates failure at the adhesive interface between the core material and the post or dentin rather than within the root. This failure mode suggests better stress distribution in Group 3 and Group 4 (Table 1).

**Table 1:** Distribution of samples according to fracture location after load application Data is represented in the form of n (%). Group 1: The control group comprised teeth with no ferrule and was restored with a metal post. Group 2: No ferrule and restored with a glass fiber post. Group 3: 2 mm ferrule and restored with a glass fiber post. Group 4: 3 mm ferrule with a glass fiber post.

Fracture location	Group 1		Group 2		Group 3		Group 4		Total	
	n	%	n	%	n	%	n	%	n	%
Vertical root fracture	14	25%	9	16%	0	0%	0	0%	23	41%
Post debonding	0	0%	5	9%	0	0%	0	0%	5	9%
Core debonding	0	0%	0	0%	12	21%	14	25%	26	46%
Coronal third of root	0	0%	0	0%	2	4%	0	0%	2	4%
Total	14	25%	14	25%	14	25%	14	25%	56	100%

The distribution of samples according to fracture type after load application revealed a pattern of fracture outcomes among the four groups. Unfavorable fractures were prevalent in Group 1 and Group 2. Group 3 and Group 4 had a significantly lower occurrence of unfavorable fractures. The results supported the earlier findings of better stress distribution in Group 3 and Group 4 (Table 2).

**Table 2:** Distribution of samples according to fracture type after load application Data is represented in the form of n (%). Group 1: The control group comprised teeth with no ferrule and was restored with a metal post. Group 2: No ferrule and restored with a glass fiber post. Group 3: 2 mm ferrule and restored with a glass fiber post. Group 4: 3 mm ferrule with a glass fiber post.

Fracture type	Group 1		Group 2		Group 3		Group 4		Total	
	n	%	n	%	n	%	n	%	n	%
Unfavorable	14	25%	14	25%	2	4%	0	0%	30	54%
Favorable	0	0%	0	0%	12	21%	14	25%	26	46%
Total	14	25%	14	25%	14	25%	14	25%	56	100%

Group 1 demonstrated a mean fracture resistance of 559.68 ± 92.52. Group 2 had the lowest mean fracture resistance of 215.60 ± 70.79. Group 3 exhibited a mean fracture resistance of 428.53 ± 154.27. Group 4 showed a mean fracture resistance of 439.06 ± 118.83. Group 1 demonstrated the highest fracture resistance, likely due to the rigidity of the metal post. Group 2 showed the lowest resistance, indicating inadequate reinforcement without a ferrule. Group 3 and Group 4, with ferrules, exhibited significantly improved resistance, emphasizing the ferrule’s crucial role in enhancing structural integrity and stress distribution (Table 3).

**Table 3:** Intergroup comparison of fracture resistance with Kruskal-Wallis test. Data is represented in the form of mean and SD. Group 1: The control group comprised teeth with no ferrule and was restored with a metal post. Group 2: No ferrule and restored with a glass fiber post. Group 3: 2 mm ferrule and restored with a glass fiber post. Group 4: 3 mm ferrule with a glass fiber post. * p-value <0.05: significant SD: standard deviation, CI: confidence interval

Group	95% CI for mean	Mean	SD	Chi-square value	p-value
Group 1	520.86-602.28	559.68	92.52	29.36	0.001*
Group 2	279.30-408.70	215.60	70.79		
Group 3	488.44-565.85	428.53	154.27		
Group 4	793.01-877.42	439.06	118.83		

The post-hoc analysis revealed significant differences in fracture resistance among the groups. Group 1 exhibited the highest fracture resistance, significantly outperforming Group 2 by 344.08 N, Group 3 by 131.15 N, and Group 4 by 120.62 N. Group 2 had the lowest fracture resistance, significantly lower than Group 3 by 212.93 N, and Group 4 by 223.46 N. However, no significant difference was observed between Group 3 and Group 4, suggesting that a 2 mm or 3 mm ferrule provided similar reinforcement. These findings emphasize that the ferrule effect improves fracture resistance, whereas the absence of a ferrule weakens the restoration (Table 4).

**Table 4:** Post-hoc analysis using Dunn test for pairwise comparison of groups Group 1: The control group comprised teeth with no ferrule and was restored with a metal post. Group 2: No ferrule and restored with a glass fiber post. Group 3: 2 mm ferrule and restored with a glass fiber post. Group 4: 3 mm ferrule with a glass fiber post. *p-value <0.05: significant

Pairwise	Mean difference	Standard error	U-value	p-value
Group 1 - Group 2	344.08	31.267	0.00	0.001*
Group 1 - Group 3	131.15	31.267	138.00	0.004*
Group 1 - Group 4	120.62	31.267	33.00	0.021*
Group 2 - Group 3	-212.93	31.267	21.00	0.001*
Group 2 - Group 4	-223.46	31.267	12.00	0.001*
Group 3 - Group 4	-10.53	31.267	111.00	0.950

## Discussion

The findings of this study demonstrate that the type of post-and-core system and the presence of a ferrule significantly influence the fracture resistance and failure mode of the ETT. In this study, core debonding was the predominant failure mode in Group 3 and Group 4 with prefabricated glass fiber posts and 2 mm and 3 mm ferrules, respectively. In contrast, root fractures were the primary failure mode in Group 1 with cast metal post-and-core without ferrules. These differences in failure modes can be attributed to variations in the post-material properties, stress distribution, and the presence or absence of a ferrule effect.

The fracture resistance results in our study showed that the amount of residual crown structure, as dictated by the ferrule height, significantly increased the fracture resistance of the ETT. This finding was supported by previous studies [16,17] and in contrast to some studies [18,19]. The disparity in the results could have been due to the use of posts of different materials, as de Oliveira et al. [18] used a carbon post, and al-Hazaimeh and Gutteridge [19] used a metal para post. The ferrule effect refers to a 360° collar in the tooth structure surrounding the post and core. This structure reinforces the remaining tooth by providing a protective band that resists functional and lateral forces, enhances the bonding of the crown, reduces the risk of microleakage and dislodgement, and reduces stress at the post-dentin interface [20]. Glass fiber posts have an elastic modulus closer to that of dentin. This similarity allows them to absorb and distribute occlusal forces more evenly, thus reducing stress concentrations in the root [17]. Thus, combining a glass fiber post and a well-prepared ferrule might have led to better stress distribution and increased fracture resistance, as observed in our study. Group 1 (metal post) observed the highest fracture resistance due to the stainless steel alloy's higher strength and high modulus of elasticity [3,10].

The most common failure mode in the prefabricated post groups was fracture of the composite core material or post debonding, which aligns with findings from previous studies suggesting that glass fiber posts exhibit a more favorable failure mode than metal posts [21,22]. The direct technique using prefabricated glass fiber posts and composite resin cores provides a more flexible and shock-absorbing interface, allowing stress to be dispersed throughout the post-dentin interface, thereby preventing catastrophic root fractures. However, the adhesive interface between the glass fiber post, composite core, and dentin is weak, making debonding or core fracturing the primary failure mode. This study supports previous research indicating that composite resin fractures at a lower force than required to induce root fractures, making it a more conservative tooth-failure mode [23].

In contrast, Group 1 exhibited the highest fracture resistance but predominantly unfavorable failures, including vertical root fractures. The high modulus of elasticity of stainless steel cast metal posts creates stress concentration at the post-dentin interface, leading to brittle fractures of the root dentin [24]. Metal posts' rigid and non-resilient nature prevents stress absorption, causing cracks to propagate into the root structure. Figueiredo et al. [25] systematically compared root fractures between metal and glass fiber posts. They concluded that prefabricated metal posts led to a two-fold increase in root fractures compared to glass fiber posts.

The presence of a ferrule significantly influenced fracture patterns, with Group 3 and Group 4 showing predominantly favorable fractures. A ferrule functions as a mechanical bracing effect, reinforcing the remaining tooth structure and redistributing occlusal forces more effectively [20]. Group 1, which lacked a ferrule, showed predominantly unfavorable fractures, further highlighting the protective role of ferrules. The height and width of the ferrite directly affected the force distribution in the ETT. A minimum ferrule height of 1.5-2 mm is recommended [12] because it significantly enhances the fracture resistance and retention of the post-and-core system. The mechanism of action of the ferrule is to reduce the stress concentration at the core-dentin interface and prevent leverage forces that contribute to post-dislodgment or root fractures. Group 2 (glass fiber post without ferrule) exhibited the lowest fracture resistance because the absence of a ferrule weakened the coronal dentin support, leading to early failure under load.

The forces experienced by the ETT vary significantly based on functional and parafunctional loading. Lyons and Baxendale [26] reported that the mean occlusal force applied to a maxillary canine was 215 N, increasing to 254.8 N under parafunctional loading, with maximum forces ranging from 343 to 362.6 N. Given these values, the results of this study suggest that teeth restored with glass fiber posts and a ferrule can withstand functional occlusal forces but may still fail under excessive parafunctional forces. In contrast, metal post-restored teeth can withstand higher forces but are at a greater risk of catastrophic failure.

AH Plus sealer (Dentsply Sirona, Germany) was used in this study and preferred over other endodontic sealers due to its superior physical, chemical, and biological properties. It offers excellent sealing ability with low shrinkage and high dimensional stability, minimizing the risk of microleakage. Its epoxy resin composition ensures strong adhesion to dentin, providing long-term sealing. Additionally, AH Plus exhibits good biocompatibility with low cytotoxicity, making it safer for periapical tissues. It also has antimicrobial properties due to the initial release of formaldehyde, reducing bacterial contamination within the root canal system. With an extended working time, it allows flexibility during obturation procedures. Furthermore, its high radiopacity makes it easily detectable in radiographs for post-treatment evaluation. Unlike traditional sealers that may dissolve or degrade over time, AH Plus remains stable and resistant to dissolution, ensuring long-term effectiveness. These advantages make AH Plus a reliable and widely used sealer in endodontic treatments [27].

Clinical implications of the study

Metal posts offer high fracture resistance but increase the risk of non-repairable root fractures, making them less favorable in structurally compromised teeth. Prefabricated glass fiber posts with composite resin cores exhibit lower fracture resistance but produce more favorable repairable failures, making them clinically preferable. The presence of a ferrule significantly enhances the fracture resistance, reduces the stress concentration, and prevents core debonding or post-dislodgment. A minimum ferrule height of 2 mm is recommended to optimize the retention and resistance forms in post-retention restorations.

Limitations of the study

The in vitro study design may not entirely emulate the intricate oral conditions encompassing dynamic occlusal forces, humidity, and temperature fluctuations that affect long-term restorative efficacy. Subsequently, disparities in tooth morphology and dentin integrity, which can influence fracture resilience, have not been addressed. Moreover, the study employed a single load application, which did not precisely replicate the cyclic fatigue loading observed in clinical scenarios. Ultimately, while metal and glass fiber posts were compared, alternative modern post materials, such as ceramics or carbon fiber, were not evaluated. Prospective investigations should integrate clinical trials and fatigue assessment.

## Conclusions

This study underscores the crucial role of ferrule height in enhancing fracture resistance and influencing failure patterns in ETT restored with post-and-core systems. Although metal posts provide higher fracture resistance, they result in unfavorable root fractures. In contrast, glass fiber posts exhibited more favorable and repairable failure modes, particularly when combined with a ferrule. A minimum ferrule height of 2 mm significantly improved the fracture resistance and reduced post-debonding and core failures. Clinically, glass fiber posts with adequate ferrule heights are recommended to preserve tooth integrity and ensure long-term restoration success, highlighting the importance of meticulous treatment planning.
